# Rodent Abundance and Hantavirus Infection in Protected Area, East-Central Argentina

**DOI:** 10.3201/eid2401.171372

**Published:** 2018-01

**Authors:** Malena Maroli, María Victoria Vadell, Paula Padula, Isabel E. Gómez Villafañe

**Affiliations:** Centro de Investigaciones Científicas y Transferencia de Tecnología a Producción, Diamante, Argentina (M. Maroli);; Universidad de San Martín, San Martin, Argentina (M.V. Vadell);; Instituto Nacional de Enfermedades Infecciosas Administración Nacional de Laboratorios e Institutos de Salud Dr. Carlos G. Malbrán, Buenos Aires, Argentina (P. Padula);; Consejo Nacional de Investigaciones Científicas y Técnicas, Buenos Aires (I.E. Gómez Villafañe);; Universidad de Buenos Aires, Buenos Aires (I.E. Gómez Villafañe)

**Keywords:** hantavirus, viruses, infection, climate, weather, disease transmission, ecology, environmental factors, rodents, Akodon azarae, grass mouse, rodent abundance, logistic regression models, antibody prevalence, zoonoses, protected area, Otamendi Natural Reserve, Argentina

## Abstract

We captured 3 hantavirus rodent hosts in Otamendi Natural Reserve, Argentina, during 2007–2012. Hantavirus antibodies were found only in *Akodon azarae* grass mice, mainly in males and old animals. Higher abundance of this species was associated with warm and rainy weather and high water levels, which peaked after a strong El Niño event.

Hantavirus pulmonary syndrome is an emerging infectious disease caused by New World hantaviruses (family *Hantaviridae*) and transmitted by rodents of the family *Cricetidae* (*1*). In Argentina, 7 native rodent species have been identified as hantavirus reservoirs (*2*). Three of these species (*Oligoryzomys flavescens* [yellow pigmy rice rat], host of Lechiguanas virus; *O. nigripes* [black-footed pigmy rice rat], host of Juquitiba virus; and *Akodon azarae* [grass mouse], host of Pergamino virus) are present in east-central Argentina. *A. azarae* mice have not been associated with cases of hantavirus pulmonary syndrome (*2*). The purpose of this long-term study was to identify factors affecting hantavirus infection and reservoir abundance.

## The Study

We conducted a study in 6 habitats in the Otamendi Natural Reserve (34°10′S, 58°48′W); (Technical Appendix) in Buenos Aires, Argentina, an area with low anthropogenic and no rodenticide pressures. Rodents were live-trapped during September 2007–December 2012. During December 2011–December 2012, for logistic and security reasons, trapping was concentrated in lowlands, salty grasslands, and highlands containing *Ligustrum* spp. Traps were baited with a mixture of peanut butter, fat, and rolled oats, placed every 10 m on permanent grids or transects, depending on the shape of each habitat, and set for 3 consecutive nights.

We ear-tagged each captured rodent; identified its species, breeding status, body length (an indicator of age) and body mass; and obtained a blood sample from a cut on the tip of the tail to test for hantavirus antibody (*3*). Rodents were released at point of capture. We calculated trap success (number of captured rodents/number of trap-nights), species richness, abundance ratio (TS_i_/TS_total_), the Shannon-Weaver Diversity Index, and hantavirus antibody prevalence by species, habitat, and trap session. We calculated body condition as weight divided by the cube of body length.

We recorded percentages of green/dry grass and broadleaf cover 1 m in height, bare ground, and maximum vegetation height during April 2009–December 2012 by using a 1-m^2^ quadrant placed around each trap station (*4*). We calculated maximum and minimum temperatures, monthly rainfall, and number of days with temperatures <0°C during the month before each trapping session. These variables were also used with time lags of 1 and 2 months. We determined anomalies in temperature by using the Oceanic Niño Index (*5*). We recorded mean, maximum, and minimum water levels in the Paraná River during the month before each trapping session and the number of months since the last flooding event for each trapping session.

We assessed associations between hantavirus infection (estimated as antibody prevalence) and vegetation, hydrologic, meteorologic, and rodent population characteristics; presence of hantavirus antibody and individual characteristics; and known hantavirus host abundanc, and vegetation, hydrologic, and meteorologic characteristics. Analyses were conducted by using logistic regressions with forward-stepping selection and binomial family distributions of errors and logit link or clog-log functions (*6*) in R software (multcomp and car packages) (*7*).

**Figure 1:**
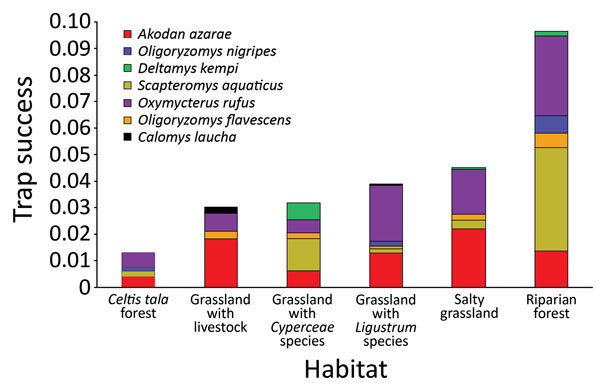
Trap success (average no. animal captures/trap-night) by rodent species in each habitat of Otamendi Natural Reserve, Argentina, 2007–2012. *Cavia aperea* Brazilian guinea pigs and *Holochilus* sp. marsh rats were not included because too few animals were captured.

During the study period, we captured 650 animals 752 times during 15,833 trap-nights. We captured 3 known hantavirus rodent host species: *A. azarae* grass mice (n = 204), *O. flavescens* yellow pigmy rice rats (n = 36) and *O. nigripes* black-footed pigmy rice rats (n = 20). We also captured 6 other species: *Oxymycterus rufus* red hocicudos (n = 223), *Scapteromys aquaticus* swamp rats (n = 129), *Deltamys kempi* Kemp grass mice (n = 27), *Calomys laucha* small vesper mice (n = 7), *Cavia aperea* Brazilian guinea pigs (n = 3), and *Holochilus* sp. marsh rats (n = 1) (Figure 1).

We detected hantavirus antibodies in *A. azarae* grass mice (Table 1) and in 1 *D. kempi* Kemp grass mouse. In the study area, *A. azarae* grass mice are the known reservoirs of Pergamino virus, a hantavirus variant that has not been associated with human disease (*2**,**8*). However, because virus mutations can result in infection of other hosts, constant monitoring is needed. Antibodies in the *D. kempi* Kemp grass mouse were probably caused by a spillover event (*2*). Maroli et al. (*9*) demonstrated that *A. azarae* grass mice share regions with other rodents, suggesting that these mice could promote spillover infections. Antibody prevalence in *A. azarae* grass mice was 23.9%, which exceeded prevalences reported in other areas of the Argentinean pampas (*10*).

**Table 1:** Characteristics of *Akodon azarae* grass mice per habitat in Otamendi Natural Reserve, Argentina

Habitat	*A. azarae* trap success rate (no.)*	Males, %	Body length, mm	Hanatavirus antibody prevalence, %
Riparian forest	0.014 (23)	70	102	35
*Celtis tala* forest	0.004 (8)	63	99	25
Lowland grassland with *Cyperaceae*	0.006 (22)	59	103	35
Highland grassland with *Ligustrum* spp.	0.013 (44)	61	93	2
Highland grassland with livestock	0.018 (32)	54	99	28
Salty grassland	0.022 (75)	61	99	29

*Values in parentheses are total number of *A. azarae* grass mice.

We captured antibody-positive rodents at every site (Table 1; Technical Appendix Figure 2) but results showed no evidence of spatial focality, in contrast to what has been reported for other hantavirus–rodent systems (*11**,**12*). Significantly greater prevalence was associated with low green grass cover (estimate −0.03721, p = 0.050). Although variation in antibody prevalence among seasons was not significant, absence of continuous trapping of antibody-positive rodents throughout the study (Technical Appendix Figure 2) suggests that temporary local virus extinctions might be a factor in some local populations, which would later have virus reintroduced from nearby source populations (*12*). However, these results should be interpreted cautiously because they might be caused by a failure to detect low levels of antibody in these populations.

**Figure 2:**
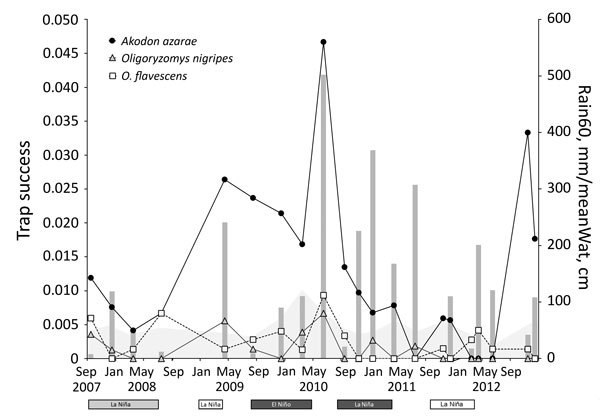
Trap success (average no. animal captures/trap-night) of known hantavirus rodent host species in Otamendi Natural Reserve, Argentina (lines), monthly accumulated rainfall applying time lags of 2 months (Rain60; gray bars), and mean water level during the month before each trapping session (MeanWat, gray shaded area), September 2007–December 2012. El Niño and La Niña events and their intensities (white, weak; light gray, moderate; and dark gray, strong) are shown below the x-axis.

The effect of community characteristics on disease risk is a topic of current debate (*13*). Hantavirus antibody prevalence was not associated with any of the community variables analyzed. However, this lack of association with environmental variables cannot be considered conclusive because of difficulties in assessing the role of environmental factors in such a complex system.

Antibody-positive *A. azarae* grass mice were more likely to have a longer body length (mean 106 mm, estimate 0.0929; p<0.001) (Table 1) than antibody-negative mice (mean length 98 mm), suggesting that hantavirus transmission among rodents is primarily horizontal, as reported for many hantavirus–reservoir systems (*1*). In addition, male *A. azarae* grass mice (estimate 1.4440; p = 0.0027) (Table 1) were more frequently infected than female mice, probably because of aggressive encounters with other rodents (*1*). Maroli et al. (*9*) reported that longer *A. azarae* grass mice travel greater distances, increasing the probability of intraspecific encounters and potential hantavirus transmission.

Abundance of hantavirus host species in the Otamendi Natural Reserve was generally associated with warm and rainy weather and high water levels; abundance was highest after a strong El Niño event and lowest after a strong La Niña event (Figure 2; Table 2). These variables might indirectly affect rodent population abundance as proposed in the trophic cascade hypothesis (*14*).

**Table 2:** Variation in abundance of 3 rodent species in relation to environmental characteristics in Otamendi Natural Reserve, Argentina*

Explanatory variable	*Akodon azarae *grass mouse				*Oligoryzomys nigripes *black-footed pigmy rice rat				*O. flavescens *yellow pigmy rice rat		
	Estimate	SE	p value		Estimate	SE	p value		Estimate	SE	p value
Meteorological model	(28.90%)				(35.50%)				(15.45%)		
Intercept (riparian forest)	−2.937^bc^	0.604	<0.001		6.033^a^	0.435	<0.001		−1.586	1.217	0.193
* Celtis tala* forest	−1.426^e^	0.415	<0.001		−1.972^b^	0.678	0.004		–	–	
Lowland grassland with *Cyperaceae *spp.	−0.923^de^	0.302	0.002		–	–	–		−0.962	0.489	0.049
Highland grassland with *Ligustrum* spp.	−0.161^cd^	0.261	0.536		−1.555^b^	0.520	0.003		−1.631	0.604	0.007
Highland grassland with livestock	0.169^ab^	0.278	0.543		–	–	–		−0.709	0.560	0.206
Salty grassland	0.390^a^	0.242	0.107		–	–	–		−0.932	0.489	0.057
Rain60	0.002	0.001	0.001		0.006	0.002	<0.001		–	–	–
MaxT	−0.049	0.018	0.005		–	–	–		−0.120	0.040	0.002
ONI	0.513	0.098	<0.001		0.408	0.384	0.289		0.305	0.270	0.258
Vegetation model	(12.34%)				(11.46%)				(6.37%)		
Intercept	−3.210	0.166	<0.001		−8.999	1.230	<0.001		−7.070	0.459	<0.001
Height	−0.255	0.038	<0.001		–	–	–		–	–	–
GBroad	–	–	–		−0.025	0.010	0.013		0.020	0.008	0.011
Hydrological model	(22.10%)				(16.85%)				–		
Intercept (riparian forest)	−5.289	0.282	<0.001		−6.068	0.636	<0.001		–	–	–
* Celtis tala* forest	−1.275	0.412	0.002		−1.514	0.653	0.020		–	–	–
Lowland grassland with *Cyperaceae* spp.	−0.732	0.300	0.015		–	–	–		–	–	–
Highland grassland with *Ligustrum* spp.	0.012	0.260	0.964		−1.245	0.511	0.015		–	–	–
Highland grassland with livestock	0.278	0.276	0.313		–	–	–		–	–	–
Salty grassland	0.560	0.241	0.020		–	–	–		–	–	–
MeanWat	1.149	0.199	<0.001		1.183	0.585	0.043		–	–	–

*The percentage of variance explained by each model is shown in parentheses in the first line above the explanatory variables. Superscript letters show significant differences in abundance among habitat types (p<0.05 by multiple Tuckey comparisons). *O. flavescens *yellow pigmy rice rats did not show differences in abundance among habitat types. Height, maximum vegetation height; GBroad, fresh broadleaf cover; MaxT, maximum temperature during the month before each trapping session; MeanWat, mean water level during the month before each trapping session; ONI, Oceanic Niño Index; Rain60, monthly accumulated rainfall applying a time lag of 2 months; –, not included in the final model.

We captured *A. azarae* grass mice in all habitats (Figure 2), showing that this species can occupy many areas and sites with low vegetation heights (Table 2). We also trapped *O. flavescens* yellow pigmy rice rats in 5 of 6 habitats but at lower rates (Figure 1; Table 2), consistent with other studies showing that this species is not dominant in the rodent community (*15*). Low abundance and short-distance movements (*9*) might restrict virus dispersal among habitats and could be the reason for the lack of detection of virus antibody. *O. nigripes* black-footed pigmy rice rats are found in many areas, (*2*), but on the basis of our study, prefer habitats with trees (Table 2). This species was also found in areas with low green broadleaf cover (Table 2).

## Conclusions

Abundance of hantavirus reservoir rodents was influenced principally by meteorologic factors that could be used to predict host population dynamics. However, the presence of hantavirus antibody was mainly influenced by rodent sex and age. Although the prevalence of infection did not vary with environmental factors, greater abundance of hosts indicates a greater absolute number of infected rodents, and therefore, an increased risk for transmission to humans.

**Technical Appendix.** Additional information on rodent abundance and hantavirus infection in protected area, East-Central Argentina.
