# Supplementary material for: Rodent Abundance and Hantavirus Infection in Protected Area, East-Central Argentina
Source: Emerg Infect Dis. 2018 Jan;24(1):131–4. doi: 10.3201/eid2401.171372 (PMC5749438; doi:10.3201/eid2401.171372)
Supplement: Supplementary file 1 — Technical Appendix. Additional information on rodent abundance and hantavirus infection in protected area, East-Central Argentina. [file 17-1372-Techapp-s1.pdf]

Article DOI: https://doi.org/10.3201/eid2401.171372

Rodent Abundance and Hantavirus Infection in Protected Area, East-Central Argentina

Technical Appendix

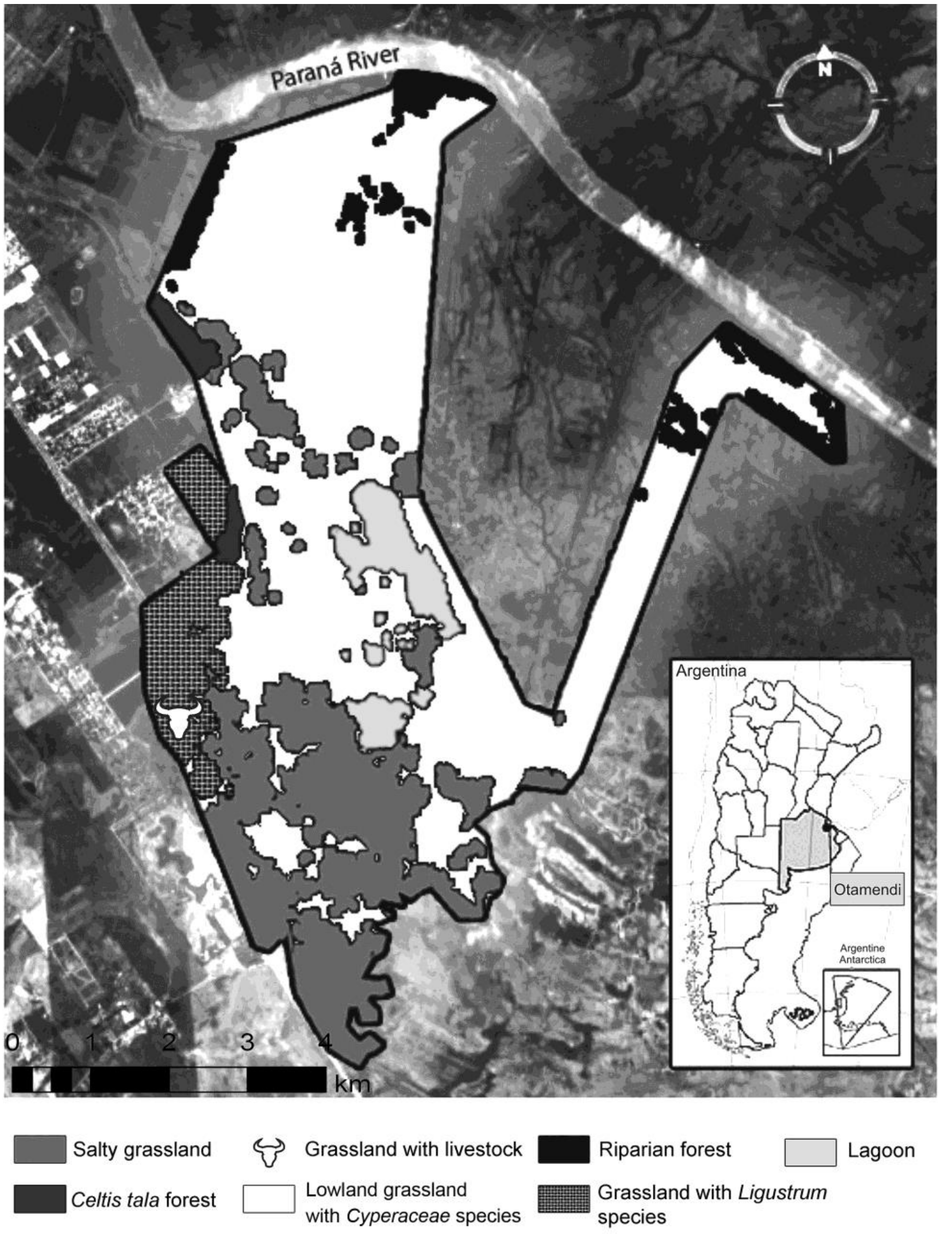

Technical Appendix Figure 1. Location of habitats studied in the Otamendi Natural Reserve, Argentina.

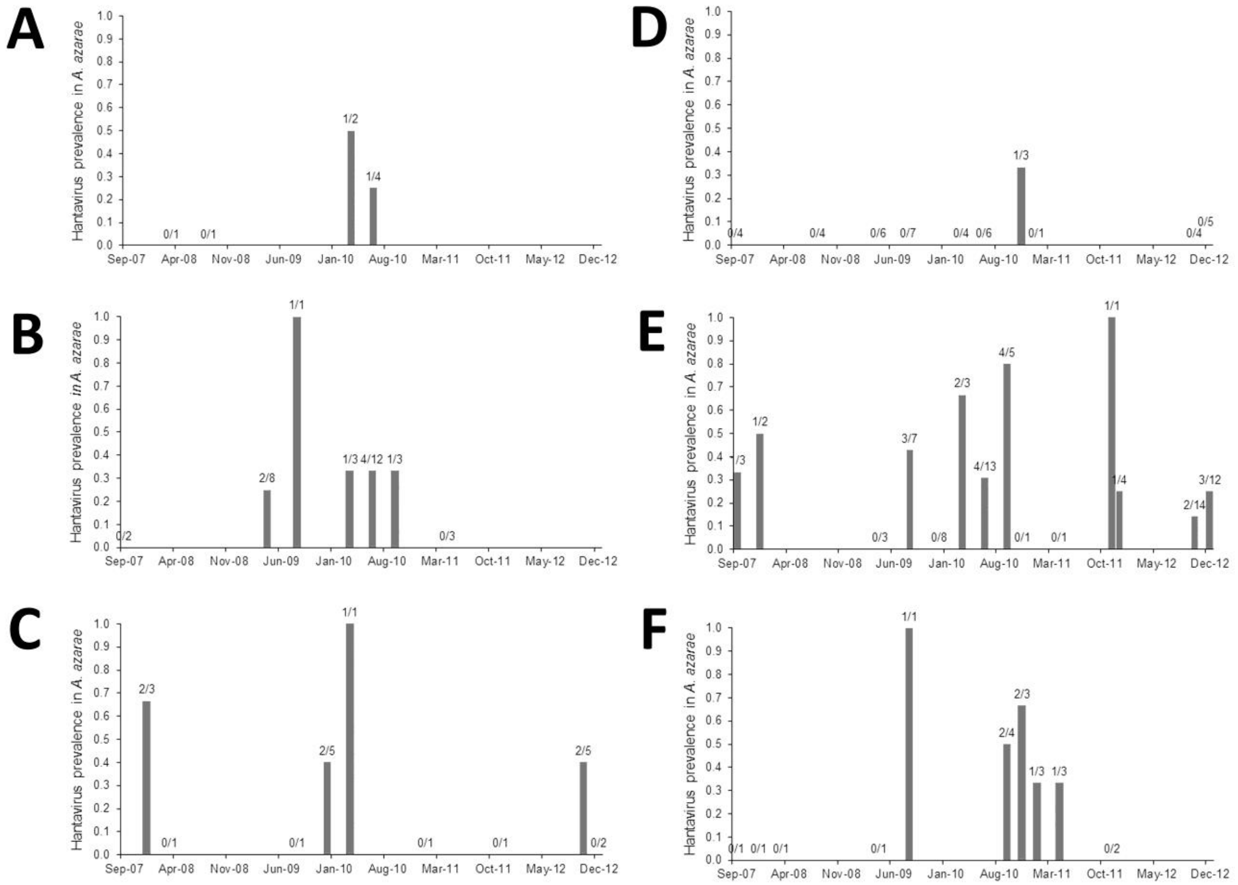

Technical Appendix Figure 2. Hantavirus prevalence in *Akodon azarae* grass mice in A) *Celtis tala* forest, B) highland grassland containing livestock, C) lowland grassland containing *Cyperaceae* species, D) highland grassland containing *Ligustrum* sp., E) salty grassland, and F) riparian forest in the Otamendi Natural Reserve, Argentina, 2007–2012. Values indicate no. positive/no. tested.
